# Development, Validation, and Comparison of a Novel Nociception/Anti-Nociception Monitor against Two Commercial Monitors in General Anesthesia

**DOI:** 10.3390/s24072031

**Published:** 2024-03-22

**Authors:** Clara M. Ionescu, Dana Copot, Erhan Yumuk, Robin De Keyser, Cristina Muresan, Isabela Roxana Birs, Ghada Ben Othman, Hamed Farbakhsh, Amani R. Ynineb, Martine Neckebroek

**Affiliations:** 1 Department of Electromechanics, System and Metal Engineering, Ghent University, 9052 Ghent, Belgium; claramihaela.ionescu@ugent.be (C.M.I.); erhan.yumuk@ugent.be (E.Y.); robain.dekeyser@ugent.be (R.D.K.); isabelaroxana.birs@ugent.be (I.R.B.); ghada.benotham@ugent.be (G.B.O.); hamed.farbakhsh@ugent.be (H.F.); amani.ynineb@ugent.be (A.R.Y.); 2 Department of Automation, Technical University Cluj-Napoca, Memorandumului Street 20, 400114 Cluj, Romania; cristina.muresan@aut.utcluj.ro; 3 Department of Control and Automation Engineering, Istanbul Technical University, Maslak, Istanbul 34469, Turkey; 4 Department of Anesthesia, Ghent University Hospital, Corneel Heymanslaan 10, 9000 Ghent, Belgium; martine.neckebroek@ugent.be

**Keywords:** modeling, general anesthesia, skin impedance, nociception index, closed-loop control, real-time monitoring, model adaptation, patient models

## Abstract

In this paper, we present the development and the validation of a novel index of nociception/anti-nociception (N/AN) based on skin impedance measurement in time and frequency domain with our prototype AnspecPro device. The primary objective of the study was to compare the Anspec-PRO device with two other commercial devices (Medasense, Medstorm). This comparison was designed to be conducted under the same conditions for the three devices. This was carried out during total intravenous anesthesia (TIVA) by investigating its outcomes related to noxious stimulus. In a carefully designed clinical protocol during general anesthesia from induction until emergence, we extract data for estimating individualized causal dynamic models between drug infusion and their monitored effect variables. Specifically, these are Propofol hypnotic drug to Bispectral index of hypnosis level and Remifentanil opioid drug to each of the three aforementioned devices. When compared, statistical analysis of the regions before and during the standardized stimulus shows consistent difference between regions for all devices and for all indices. These results suggest that the proposed methodology for data extraction and processing for AnspecPro delivers the same information as the two commercial devices.

## 1. Introduction

The burden of developing suitable sensing techniques for use in closed-loop optimization of drug management problems is becoming crucial as the medical and engineering communities are increasingly joining efforts. Among the applications of fast computing and data driven information processing for real-time extraction of individualized patient models, we propose here solutions for closed-loop control of general anesthesia [1]. In-time monitoring and reliable data processing techniques for real-time extraction of patient-related information enables closed-loop control with search algorithms for optimal drug dose management to maintain general anesthesia parameters under the surgical stimuli and other biological response closed loops [2,3,4,5]. The choice of monitoring devices is, therefore, crucial as their relevance for the closed loop can make the difference between applicability of an optimal search algorithm or the bottleneck of lack of reliable data information [6,7,8].

The general anesthesia drug management problem has already two matured and established computer-assisted closed loops: (i) for hypnosis and (ii) for neuromuscular blockade. The clinical practice uses standardized monitors for model-based computerized management of the respective drugs. The control algorithms range from anything in between feedforward, feedback, and predictive-based methodologies [9,10,11,12]. Most popular solutions in the literature are the proportional–integral–derivative (PID) control [13,14,15] and model-based predictive control (MPC) [16,17,18,19,20,21].

Advantageously, simple PID control requires a mere model approximation for initial tuning of controller parameters and can be fine-tuned to achieve acceptable performance within safe vital intervals [1,22]. The more complex MPC algorithms can apriori deal with imposed constraints, variable delays in monitored information, and give optimal solutions within the feasibility domain, and may continuously adapt controller parameters to tackle changes in a patient’s response and mitigate critical events with new imposed constraints [23,24].

There is a stringent need for reliable and fast-computing models to allow further developments of MPC applications to general anesthesia. Unlike the aforementioned two components, the third component of general anesthesia, namely, analgesia or anti-nociception (i.e., lack of pain), is not yet resolved in terms of monitors for closed-loop applicability, and lacks models from drug infusion rates of opioids (e.g., Remifentanil) to their effect in the patient to alleviate nociceptor stimulation effects (i.e., pain stimulation in the presence of opioid medication). A comprehensive overview of nociception/anti-nociception (N/AN) monitoring devices is given in [25] and discussed for their suitability for use in closed-loop anesthesia management, with examples of success stories in the literature. As such, two candidate commercial devices stand out: Medasense and Medstorm monitors; their performance in real clinical environment is analyzed and compared in our paper.

In this paper, we propose a carefully designed protocol of alternate monitoring devices within the standardized stages of the induction phase of general anesthesia and sequences before surgical initiation. This protocol allows us to introduce and validate the prototype AnspecPro monitoring device, compared with the use of two commercial devices Medstorm and Medasense. The data collected allow us to finalize the developing stages of the AnspecPro device into an end-user skin-impedance-based index of nociception level. The commercial devices also deliver an index of nociception level. Based on this information at hand, the usefulness and causality of each index can be analyzed with respect to the infused opioid drug and their relevance for integration within any closed-loop control methodology can be assessed. A dynamic causal model is estimated for each monitor in terms of input (drug Remifentanil) and output (index) transfer functions.

The original contributions of this paper are the following:

- Development and validation of the AnspecPro prototype device based on clinical data evaluated for N/AN level;
- Clinical-data-based models for dynamic response in patients undergoing general anesthesia for three monitors from opioid (i.e., Remifentanil) to N/AN level (index);
- Statistical analysis and comparison of monitor performance under general anesthesia.

## 2. Materials and Methods

### 2.1. Clinical Protocol and Patient Database

**Figure 1:**
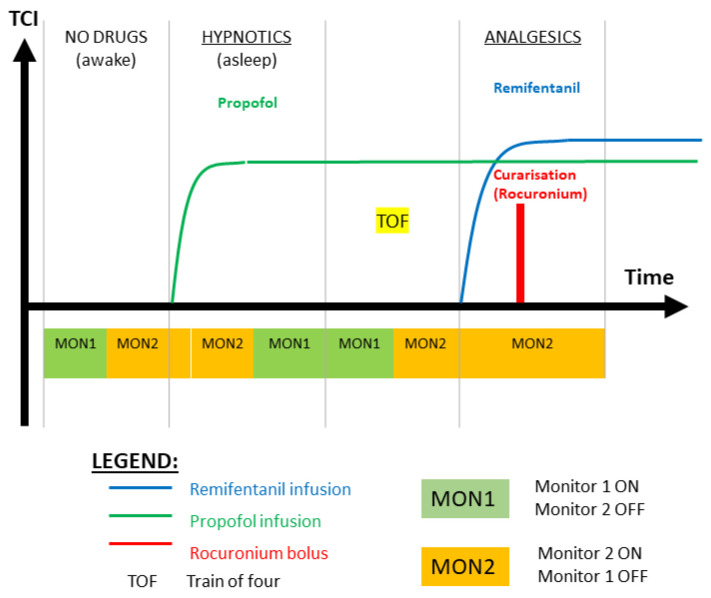
Illustration of the clinical protocol for testing monitors in pairs of two per patient per action or stage of the general anesthesia interval. MON1 and MON2 are two randomly blinded selected monitors in pairs of two per patient. See Table 1 for details per patient.

The *goal* of the clinical investigation was to validate the new N/AN AnspecPro monitor during total intravenous anesthesia (TIVA) by investigating its outcomes related to noxious stimulus. Additionally, we also compare it to two other commercial N/AN monitors: Medasense Monitor (CE0344, Manufacturer: Medasense Biometrics Ltd., Ramat Gan, Israel) and the Medstorm Monitor (CE0413, Manufacturer: Med-Storm Innovation AS, Oslo, Norway). The clinical protocol of the testing of the three monitors is depicted in Figure 1. The regions of interest presented in this paper are the Hypnotics region, where the TOF (train of four) test is applied, and the region denoted by “Analgesics” from the initial titration of Remifentanil right before intubation occurs. The data recordings analyzed in this paper are those performed with Monitor 2 in the respective region. The random selection of monitor pairs per patient and biometrics are given in Table 1. Typical data intervals in the region of interest range between 15 and 70 samples, available every 5 s. As validation of nociception stimulus, the standard test train-of-four (TOF) is applied before the Analgesics region. Here, we test the ability of all monitors to detect/react against the TOF test in their monitored N/AN index.

A total of 70 patients scheduled for elective surgery under TIVA were enrolled in the study after signing their informed consent. During the preoperative consultation and prior to entry in the trial, the investigator (the anesthesiologist) explained to potential subjects the trial and the implication of participation. Subjects were informed that their participation is voluntary and that they may withdraw consent to participate at any time. After this explanation and before entry to the trial, written, dated, and signed informed consent was obtained from each subject.

During the perioperative inclusion process, participants were randomly assigned a pain monitor using sealed envelopes to ensure blinding. A total of 180 sealed envelopes, each containing one of the three pain monitors, were prepared. These envelopes were divided into three groups, each comprising 60 participants. Participants selected an envelope from the pool of 180 (or the remaining envelopes if the study had already commenced), and, upon opening, were allocated the pain monitor enclosed within. Randomization codes were securely stored in the case report form (CRF) to maintain blinding and ensure the integrity of the randomization process.

The inclusion criteria were adults with age between 18 and 80 years, American Society of Anesthesiologists (ASA) Class I, II, and III classified by the anesthesiologist, and patients able to comprehend, sign, and date the written informed consent document to participate in the clinical trial.

Exclusion criteria were patients having epidural analgesia infused by a pain pump during the operation, patients with chronic pain or under chronic pain medication (i.e., antiepileptics, antidepressants, opioids), pregnant women (asked at the patient before the operation), and patients with electrically sensitive life support systems (e.g., implanted pacemaker, defibrillator).

This trial was conducted in accordance with the protocol, also covering ISO 14155 which addresses good clinical practices (GCP) and applicable law(s). ISO 14155 is an international ethical and scientific quality standard for designing, conducting, recording, and reporting trials that involve the participation of human subjects. Compliance with this standard provides public assurance that the rights, safety, and wellbeing of trial subjects are protected, consistent with the principles originating from the Declaration of Helsinki, while the clinical trial data are credible.

The trial for intraoperative nociception monitoring during TIVA was a randomized (1:1:1) controlled trial to validate a new N/AN monitor during general anesthesia by investigating its outcomes related to noxious stimulus. It pursues the validation of the Anspec-PRO (non-CE marked) by comparing its function with the already CE-marked monitors Medasense Monitor (CE0344, Manufacturer: Medasense Biometrics Ltd., Ramat Gan, Israel) and the Medstorm Monitor (CE0413, Manufacturer: Med-Storm Innovation AS, Oslo, Norway). The clinical investigation involving human subjects was compliant with the regulatory framework stated in the European Regulation (EU) 2017/745. This academic clinical investigation was approved by the Ethics Committee of Ghent University Hospital and the Federal Agency for Medicines and Health Products of Belgium FAGG (EC/BC-08020, FAGG/80M0840, EudraCT: CIV-BE-20-07-0342442020, clinicaltrials.gov: NCT04986163, principal investigator: Martine Neckebroek).

General anesthesia was induced and maintained by targeting desired effect-site concentrations by the anesthesiologists, with target-controlled-infusion (TCI). Initial target of effect-site concentration for Propofol was 5 μg/mL and the BIS level. The targeted effect-site concentration for Remifentanil started from 3 ng/mL, and was clinically changed by the anesthesiologist whether the level was interpreted as too high or too low (specially seen in the patient’s heart rate or blood pressure values). Muscle relaxation (curarization) was obtained with Rocuronium, guided by an induction dose of 0.3 mg/kg and titrated by additional boluses when needed, based on the clinical interpretation of the anesthesiologist.

### 2.2. Commercial Nociception/Anti-Nociception Monitors

Two commercially available and CE-marked monitors were available for this study: The Medasense Monitor (CE0344, Manufacturer: Medasense Biometrics Ltd., Ramat Gan, Israel [26,27]) and the Medstorm Monitor (CE0413, Manufacturer: Med-Storm Innovation AS, Oslo, Norway [28]).

The Medstorm monitor uses skin conductance of single-frequency testing signal and processes the number of variations in amplitude of intensity over periods of time to deliver an index between 0 and 1. This index is denoted by SC (skin conductance) and has been correlated with N/AN monitoring in various studies [29,30].

The Medasense monitor uses multivariate analysis and artificial intelligence tools on a manifold of biological signal recordings to extract features correlated with signal intensity and also delivers an index between 0–100. This index is denoted by NOL (nociception level). Several clinical studies have suggested that the device delivers a confident measure for nociception [31,32].

### 2.3. AnspecPro Prototype Monitor

The AnspecPro (AP) prototype was manufactured by the research group on Dynamical Systems and Control (UGent) for continuous monitoring by means of (noninvasive) skin impedance measurements, enabling pain/nociception measurement. It can be considered as medical device according to the definition given by the Council Directive 93/42/EEC on medical devices, repealed in 2021, after the trial approval, by the EU Regulation 2017/745 on medical devices. AnspecPro was authorized for this academic clinical investigation by the Federal Agency for Medicines and Health Products (FAGG/80M0840, 2020), the Belgian competent authority in charge of ensuring the application of MDD 93/42/EEC. The general safety and performance requirements, the design and manufacturing specifications, and the information needed to be supplied with the device were fulfilled in this trial.

AnspecPro was classified as medical device class IIa for short-term use in compliance with MDR 2017/745, taking into account the intended purpose of the device and its inherent risks. This classification includes AnspecPro in a class of devices with medium risk for the patients. The classification rule under which AnspecPro fell was as an active device for diagnosis/monitoring of vital physiological processes, as its intended use in the clinical trial is to detect the nociceptive response, in particular, noninvasive monitoring during TIVA. The electrodes and the electrode connectors are CE-marked (according to MDD93/42/EEC). The National Instruments CompactRIO that is used to measure the patient fulfills the required electrical equipment safety standards.

Following successful validation in silico [33], the AP device was validated for its sensitivity to detect variations in skin impedance time and frequency information in awake, communicative, healthy volunteers [34,35] and in clinical trials with awake and communicative postoperative care unit patients against golden standard numerical pain rating scale [35].

Once tested in an experimental setup on awake healthy subjects having mechanically induced acute pain [34], the AnspecPro monitor has also been successfully validated to detect clinical postsurgical pain [35]. AnspecPro measures the skin impedance in response to a multisine [34]:

(1) $$u\left(t\right)=\sum_{k=1}^{29}A_{k}sin\left(\omega_{k}t\right)+\phi_{k},$$

where *A* is the signal amplitude, ω=2πf (rad/s) is the angular frequency, and ϕ is the phase (rad), with the frequency f∈ [100:50:1500] Hz linearly distributed in 29 values. Three electrodes (commercial 3 M electrodes) are placed on the palm hand of the patient. For converting the two measured time vectors (i.e., current c(t) and voltage v(t) signals) in the frequency domain, the spectral power density function with a modified averaged periodogram method is used [36]. The measured data are sampled at a frequency $F_{s}=15$ kHz. The frequency components of the current and voltage signals were returned via the fast Fourier transform (FFT) for each sequence as the cross-spectra of these signals relative to the applied excitatory input signal: $S_{VU}\left(j\omega_{k}\right)$ and $S_{CU}\left(j\omega_{k}\right)$, respectively. The vector of the complex skin impedance Z(jω) is computed every 5 s as the ratio:

(2) $$Z\left(j\omega_{k}\right)=\frac{S_{VU}\left(j\omega_{k}\right)}{S_{CU}\left(j\omega_{k}\right)},$$

with $j=\sqrt{-1}$ as the imaginary number, for the total number of samples every 5 s measurement interval and containing the frequency response function for $\omega_{k}$ the 29 excited frequencies.

### 2.4. AnspecPro Monitor: N/AN Index Development

The N/AN index is, in fact, a time (and frequency) varying value, linked to physiological complex phenomena taking place in the body and measured at the skin conductance surface level. As such, the physiological phenomena in skin impedance studies has been previously explored in electrical and material science engineering domains, with models capturing various properties in various circumstances. Here, we make use of some of the most suitable analogies to extract parametric model structures that still relate to physiology but yet are minimally complex for the objective of capturing relevant changes in skin tissue impedance as a result of opioid input in the system (in the patient).

By making use of our prior expertise in modeling physiological systems [37], we propose an analogy between the tissue-related dynamic transfer impedance properties and the electrical network circuits. The simplest yet physiologically-based is the recurrent ladder network of resistors (R) and capacitors (C), whereas the recurrent properties are explainable by specific tissue properties [37]. Specifically, a single RC-cell element in this ladder network contains pairs of pole–zero polynomials [37]. This electrical network has polynomials with roots following a recurrent placement in the complex numerical plane, exhibiting phenomena well supported by the expected diffusion pattern in biological tissues [38]. We propose this simple yet effective linear parametric model:

(3) $$M_{RC}\left(s\right)=\frac{s^{2}+2r_{R}\left(m-1\right)Rs+r_{R}\left(m-1\right)R^{2}}{s^{2}+2r_{C}\left(m-1\right)Cs+r_{C}\left(m-1\right)C^{2}},$$

with *s* as the Laplace operator, m=1…N as the number of recurrent cells, and the recurrent coefficient values of $R_{m}=R\cdot \left(m-1\right)\cdot r_{R},C_{m}=C\cdot \left(m-1\right)\cdot r_{C}$. Despite its high order, due to recurrent properties, it requires estimating only 4 unknown parameters: $R,C,r_{R},r_{C}$. In Appendix B we provide a numerical example for the model solutions. An additional unknown parameter is the number of cells existing in the ladder network, but this can be apriori fixed to limit the complexity of the model. From a dynamic transfer function point of view, the locations of the roots denoting the poles of the system are the most important when characterizing dynamical variability of tissue properties as they change their value depending on the physiological phenomena occurring in the tissue, which in turn depends on the presence/absence of drug molecules in tissue. Hence, an index may be defined as

(4) $$AP1=\frac{C}{r_{C}}$$

representing the ratio of pole placement coefficient to their recurrent augmentation. This is closely related to the analytically derived fractional-order coefficient in recurrent ladder network models with continuous fraction expansions, namely,

(5) $$N_{fo}=\frac{r_{R}}{r_{R}+r_{C}}$$

which is directly related to layered tissue properties and electrical equivalent. Furthermore, studies exist to provide data to examine the validity of this fractional order coefficient, which will be used to discuss the Results section of this paper.

Recurrent ladder networks are distributed parameter models which, via continuous fraction expansion, become a network of interlacing poles and zeros [37]. Algebraic studies [39] showed that a reduced parametric model for the ladder network would be a limited number of pole–zero interlacing pairs, represented as follows:

(6) $$M_{ZP}\left(s\right)=K\frac{\left[s^{2}+Z_{1}s+Z_{2}\right]\cdot \left[s^{2}+Z_{3}s+Z_{4}\right]\cdot \left[s^{2}+Z_{5}s+Z_{6}\right]}{\left[s^{2}+P_{1}s+P_{2}\right]\cdot \left[s^{2}+P_{3}s+P_{4}\right]\cdot \left[s^{2}+P_{5}s+P_{6}\right]}$$

exemplified for a 6th-order interlacing pairs and a scaling factor *K*. Notice the difference with model (3) is that the positions of the poles and zeros by their respective polynomial coefficients are left free to be identified. A potential indicator of changes in the dynamic properties captured by such a model is the relative ratio of pole-to-zero location. This can be expressed by the following index:

(7) $$AP2=\frac{K}{6}\cdot \frac{Z_{1}}{P_{1}}\frac{Z_{2}}{P_{2}}\frac{Z_{3}}{P_{3}}\frac{Z_{4}}{P_{4}}\frac{Z_{5}}{P_{5}}\frac{Z_{6}}{P_{6}}$$

This index is closely related to considering the distance between pole-to-zero interlacing from model (3). Notice that a 6th order is needed to capture the frequency dependent impedance as a function of time, but not all of the six ratios are required to capture the variability of this function with variations of drug infusion patterns.

Both models from (3) and (6) were identified using nonlinear least squares method, with function lsqnonlin in Matlab from Mathworks™. Initial values were reiterated for estimations until no significant improvement was obtained (usually about 2–3 iterations were sufficient). The optimization process is a subspace trust region method rooted in the interior-reflective Newton method. For this particular context, parameter lower bounds were constrained to 0, ensuring non-negativity, while no upper bounds were applied. Termination of optimization was triggered upon reaching a tolerance threshold of $10^{-8}$. Across all scenarios, the correlation coefficient between data and model estimates consistently surpassed 80%.

Specifically, skin impedance modeling characterized human skin properties by single, double Cole model [40] or 3-layered model [41]. Galvanic skin response and electrodermal activity was modeled by Cole and RC-layered models [37,42,43,44] and identification of fatigue-induced changes and skeletal muscle damage by single Cole model [45]. The combination of electrodes impedance and skin tissue impedance was characterized by a single Cole model structure [46] which we are also using here as a lumped fractional order impedance model (FOIM):

(8) $$M_{FOIM}\left(s\right)=R+\frac{K}{1+{\left(\frac{s}{p}\right)}^{\beta }}$$

where *R* denotes the gain at infinite frequencies, *K* denotes the relative gain difference between low and high frequencies, and $1/p^{\beta }$ denotes pseudo-capacitance of the constant phase element. It follows that $\tau =\sqrt[{\beta }]{1/p}$ is the relaxation time constant as per analogy to Debye materials characteristics. The relaxation time constant of the material needs to be scaled to the relative gain of the excited frequency response function, as it varies significantly from low- to mid- and high-frequency range behavior. As such, an inversely scaled index could be proposed from model (8) as follows:

(9) $$AP3=\sqrt[{\beta }]{K/p}$$

Alternatively, another index holding tissue properties could be proposed as follows:

(10) $$AP4=\frac{\beta }{p}$$

representing the fractional order of the Cole model—linked to structural changes in the tissue as a function of time and frequency, and the impedance properties of the tissue, which are changing along with the drug profiles.

To summarize, the developed indices for the AnspecPro device are defined by Equations (4), (7), (9) and (10).

### 2.5. Frequency Domain Impedance Identification Methods

The parametric models from (3) and (6) were identified using nonlinear least square method and Newton–Gaussian gradient search optimization algorithm. The identification method requires initial values, which were randomly assigned in the first step. The method was applied iteratively, updating initial values to the last estimated set of values. The number of iterations was limited to 10 steps, but, in practice, this was found to be too large (usually, no significant improvement is achieved after 3–4 steps). The Matlab function lsqnonlin was used.

Given the nonlinearity in the parameters in the FOIM model from (8), genetic algorithm optimization was used for searching optimality of the cost function, with the function in Matlab 2023 ga. It solves stochastic global search optimization problems by repeatedly modifying a population of individual solutions. The algorithm creates a random initial population, from which the next generation of children is produced at each step by selecting individuals from the current population to be parents. Over successive generations of elite retentions until stopping conditions are met, the algorithm produces the crossover, and mutation children’s optimals are set [47].

To select the most suitable solution for each patient globally, we assessed the goodness of fit (gof) for every optimal parameter vector generated throughout each genetic algorithm (GA) iteration. We identified the parameter set with the highest frequency, ensuring that the corresponding fit value was at least gof<0.1. Subsequently, this parameter set was employed to initialize the model in the online procedure for the same patient. A fit value of 0 signifies an ideal match between the data and the estimated model outputs, yielding a fitting percentage equivalent to (1−gof)×100.

### 2.6. Time Domain Transfer Function Model Structure Selection and Methods

In terms of input–output block scheme, we find that drug infusions of Remifentanil correspond to a change in the drug concentration in tissue, affecting their effect. Pharmacokinetic models from compartmental modeling were not possible to develop here due to lack of data and lack of identification-rich information signals measured from the patient during general anesthesia. Instead, simplified transfer function models denoting the central compartment where the drug is applied intravenously (blood compartment) and an effect-side compartment (where the drug takes effect in the nervous system) were possible to identify. This included the gain corresponding to the pharmacodynamic models from effect site concentrations of the drugs to their effect (i.e., Hill curves).

**Figure 2:**
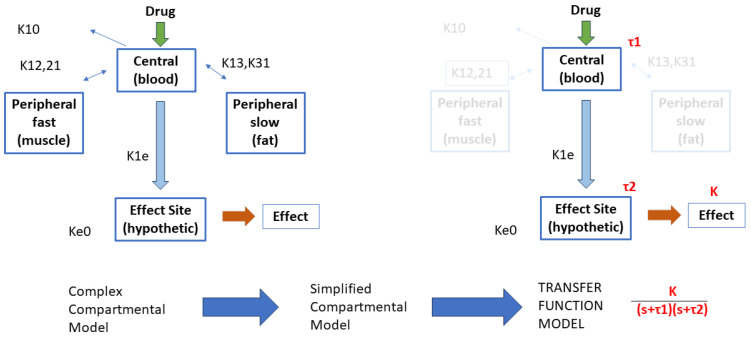
Illustration of the rationale behind the development of the simplified transfer function model structure.

A schematic flowchart is given in Figure 2. In this schematic, the variables Kij denote the kinetic rate transfer in and out of the compartments and their clearances. The simplified compartment model is then considered as a first-order transfer function with time constant τ1 for central, respectively, τ2 for effect site compartment. The nonlinear Hill curve relating the effect site drug concentration to its effect in the body of the patient is replaced by a gain coefficient *K*. The dynamic response of this transfer function will be that of a second-order damped system, i.e., having two real poles as roots in the complex plane.

For each value of the indices defined by relations (4), (7), (9), and (10), we have a corresponding infused drug rate of Remifentanil, as described in Figure 1. This provides an input–output vector relationship for the identification task. The Toolbox IDENT for system identification in Matlab from Mathworks™was used here along with the transfer function estimator command tfest.

### 2.7. Statistical Analysis

One-way analysis of variance was applied using Matlab function anova1. It gives a boxplot representation of median, quartiles’ percent, and outliers for groups of samples with normal distribution. Distribution tests, such as Anderson–Darling and one-sample Kolmogorov–Smirnov, were used to verify the sample distribution. The classification results were considered significant if p≤0.05 (i.e., within 95% confidence interval) [48]. The results of this analysis can be found in the Supplemental Material.

Similarity among signals was verified using the correlation coefficient between two signals, and was calculated using the Matlab function corrcoef. Following textbook standards in signal processing, a correlation coefficient above 0.3 was considered relevant to pursue parametric models in transfer function structure [49]. The Granger causality test was performed using both function iscausal and gctest available in Matlab environment.

Student’s *t*-tests with unequal sample size were used to compute the confidence intervals. The classification results were considered significant if p≤0.05 (i.e., within 95% confidence interval).

## 3. Results

**Figure 3:**
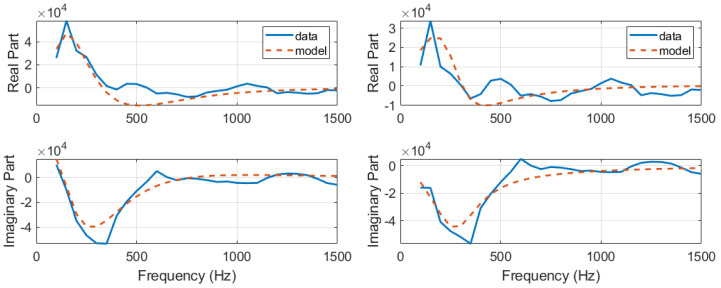
An example of identification result for the recurrent ladder network with 13 cells of second order, i.e., 26th-order polynomials (**left**) and an example of identification result for the zero-pole interlacing of 6th order (**right**).

**Figure 4:**
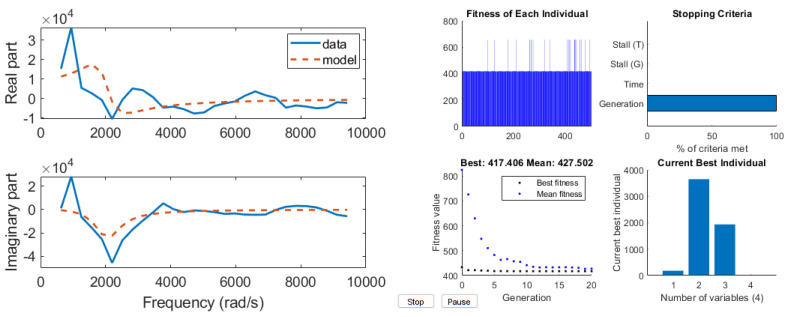
An example of identification result for the compact FOIM model in 4 parameters (**left**) and its genetic algorithm optimization solver view (**right**).

First, the development of the AnspecPro N/AN index is presented in terms of results of the identified frequency domain impedance measured at skin level, and the parametric models from Section 2.4. An example of frequency domain impedance data identification for the AnspecPro monitor in patient 24 is given in Figure 3 (left) for the model from (3). The results for the same data for the model (6) are given in Figure 3 (right), and for the model (8) in Figure 4, respectively. Indeed, as expected, the best fit is obtained by the highest-order model, namely, the model from (3) identified with 13 cells in the network, resulting in a 26th order polynomial. This impedance is available for identification every 5 s; hence, a model is estimated every 5 s during the “Analgesics” region of interest (see protocol Figure 1).

**Figure 5:**
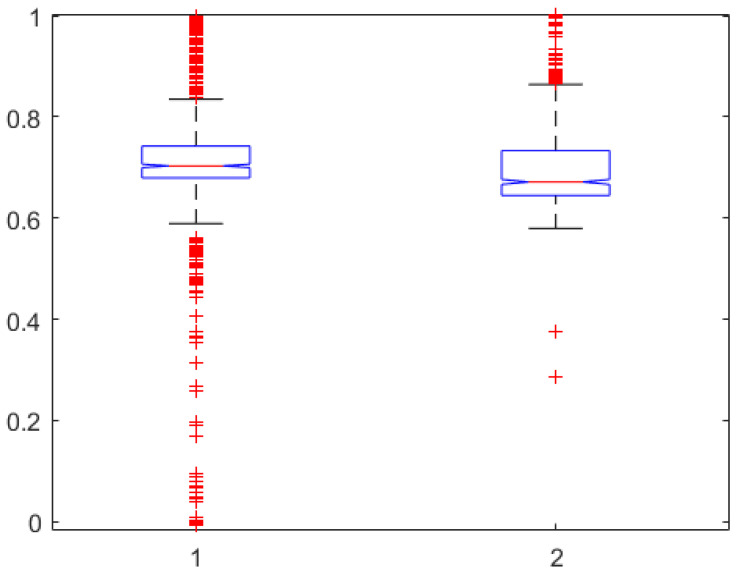
One-way ANOVA for the fractional orders calculated with (5) in the left column against the fractional order identified of β−0.942 from model (8) in the right column (p=0.61).

In terms of fractal dynamic component captured in our models, we have the relation calculated with (5). Since the corresponding lumped model of (3) is the FOIM Cole model from (8), we can look at the values of the fractional order β. However, in [46], it is discussed that fractional order values of 0.942 are accounting for the electrode effect alone. One-way ANOVA was performed on these two variables for the entire pool of data from the AnspecPro monitor. After correcting values for effect of electrode in β, no statistically significant difference is observed in Figure 5. The number of outliers is rather large given the small dataset (n=23 patients).

**Figure 6:**
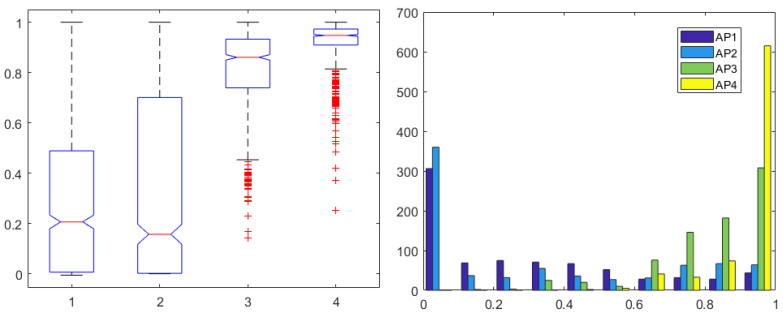
One-way ANOVA for the AP1 in first, AP2 in second, AP3 in third, and AP4 in fourth column (**left**) and histogram (**right**) for all 4 AP indices normalized for each patient.

Since the patient variability is large in terms of skin and tissue impedance values, one may not compare values directly among patients. It is therefore advisable to normalize the AP values calculated to their maximum value in each corresponding patient. In this way, all patients will have AP index values between 0–1, allowing a fair comparison. The derived four AP indices are given in Figure 6 with normalized values, along with their corresponding histogram distributions. The index from AP2 required only the second and fourth pair to be calculated, as the other pairs were insensitive to variations correlated to drug profiles. The histogram illustrates that AP1 uses a larger range of values than AP3 and AP4, also confirmed by the one-way ANOVA analysis, where no significant differences were observed (p=0.52).

**Figure 7:**
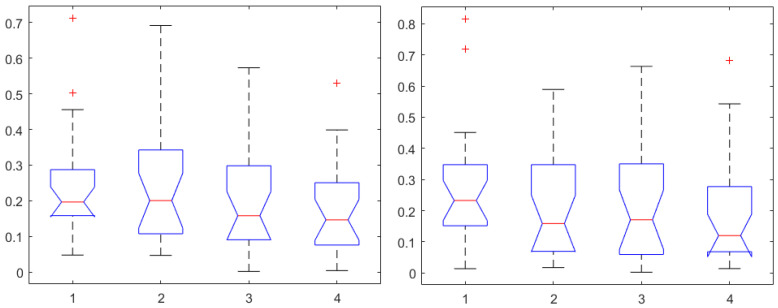
One-way ANOVA for the AP1 in first, AP2 in second, AP3 in third, and AP4 in the fourth column for correlation coefficients to Remifentanil (**left**) and to Propofol (**right**).

Secondly, to determine whether or not a relation exists between the input of the system (i.e., Remifentanil) and output of the system (i.e., N/AN index), correlation and causality analysis was performed. When identifying dynamic models such as transfer functions, one implies existence of causality. The Granger causality test was used and corresponding correlations are given in Figure 7 (left) for Remifentanil, and in Figure 7 (right) for Propofol infusion rates, respectively. As expected, the correlations to Remifentanil were slightly higher than those to Propofol, but there were no statistically significant differences observed (p<0.5). It is difficult to speculate which one of the derived indices is best suited for selection to pursue dynamic transfer function input–output model identification for the purposes of control.

**Figure 8:**
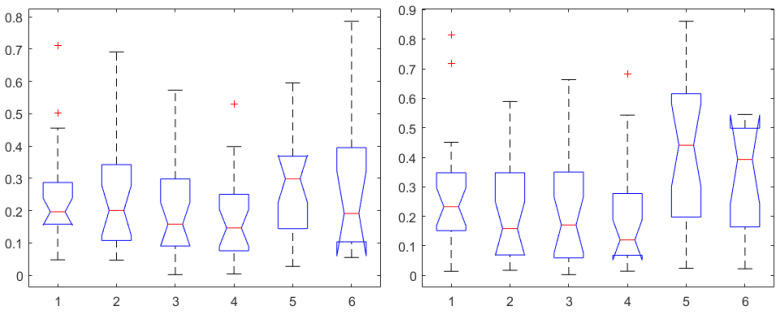
One-way ANOVA for the AP1 in the first, AP2 in the second, AP3 in the third, AP4 in the fourth, NOL in the fifth, and SC in the sixth column. Correlation coefficients for Remifentanil (**left**) and for Propofol (**right**).

However, it is interesting to compare the AP indices herein derived against the NOL and SC indices from the commercial monitors. One-way ANOVA for correlations to Remifentanil are given in Figure 8 (left), and, respectively, for Propofol in Figure 8 (right). In both cases, a statistically significant difference was determined (p<0.002) with the NOL values providing the highest correlation values, with median above 0.3 threshold. There were no statistically significant differences observed in one-way ANOVA between NOL and SC correlations to Remifentanil (p<0.77), or in correlations to Propofol (p<0.17). The results from Figure 8 suggest that NOL may be the best-suited index for building dynamic transfer function models between input infusion rates of Remifentanil to its output effect in general anesthesia.

Following the correlation test, we pursued the causality test, reported in Table 2. The lowest percent causality was obtained for the Medstorm device, whereas similar percents were obtained for the Medasense and Anspec Pro devices.

**Figure 9:**
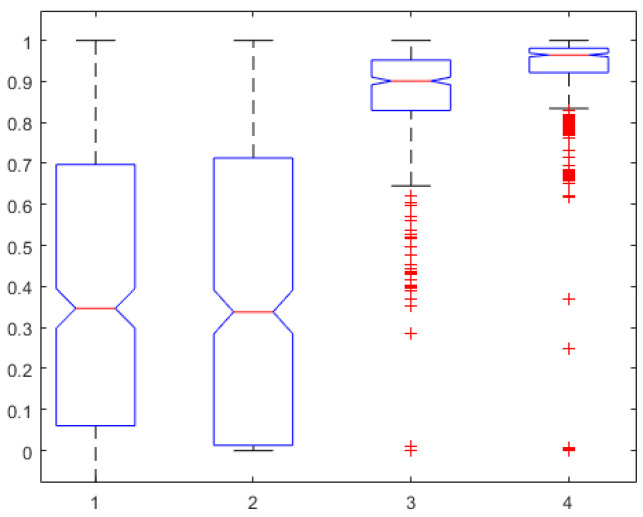
TOF region test: One-way ANOVA for the AP1 in the first, AP2 in the second, AP3 in the third, and AP4 in the fourth column normalized for each patient.

To further validate the AnspecPro monitor for noxious stimuli, we examined the data in the pre-“Analgesic” region where TOF was applied. From Figure 9, we conclude that the AnspecPro monitor is sensitive to changes in skin impedance induced by the TOF noxious stimulus, with a relatively small difference between the indices (p<0.162). More details can be found in the Supplementary Material.

For those patients where both correlation and causality conditions exist, it infers that a parametric model characterization can be obtained from the deterministic input–output signals.

**Figure 10:**
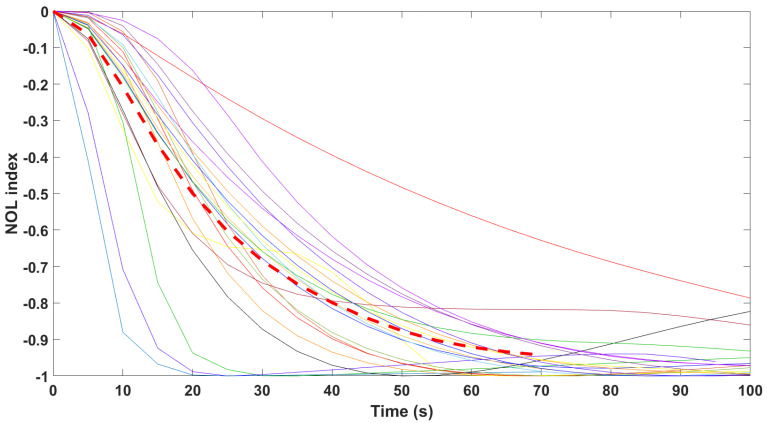
Normalized step responses for all transfer function models identified from patients evaluated with the Medasense monitor in the Analgesic region.

**Figure 11:**
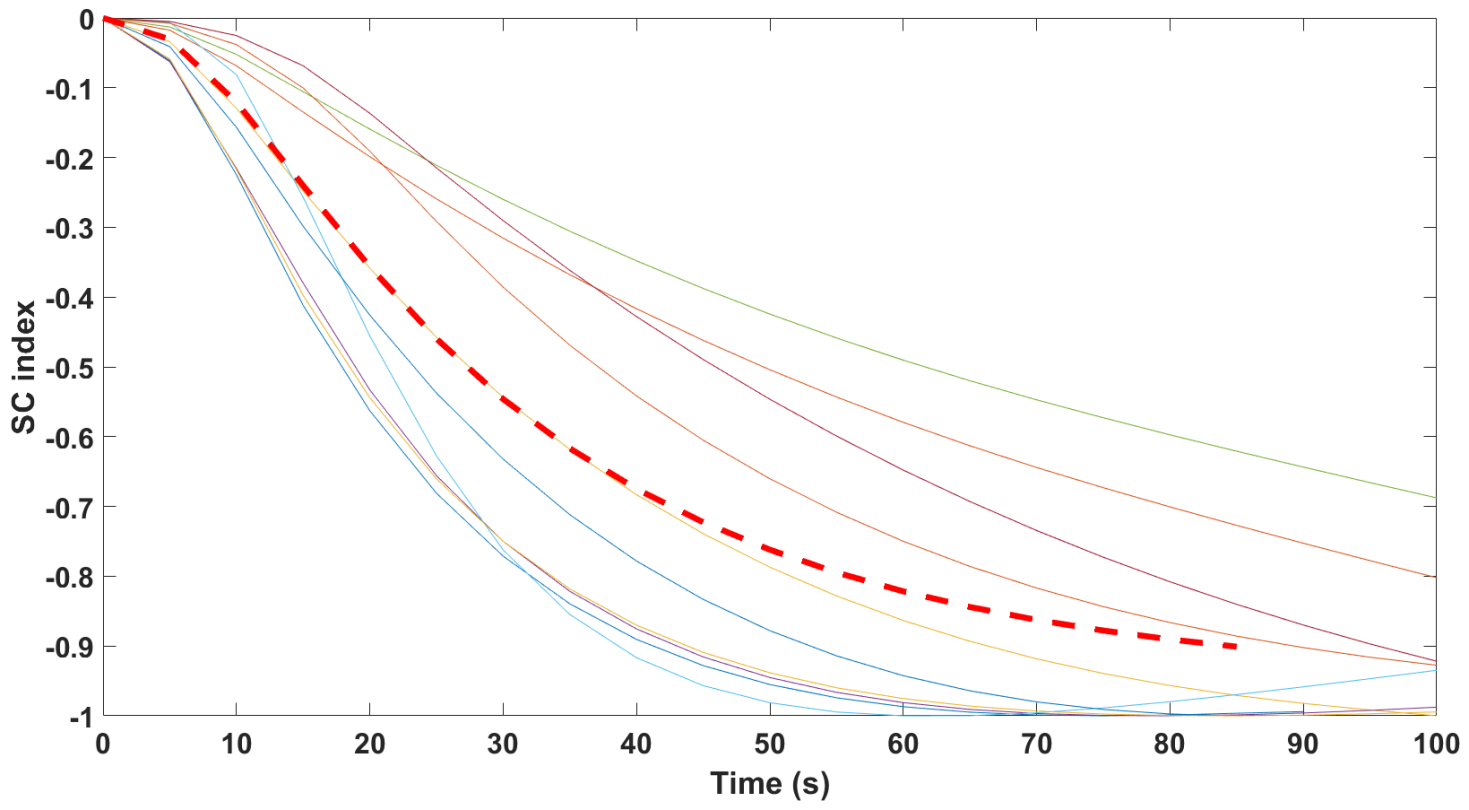
Normalized step responses for all transfer function models identified from patients evaluated with the Medstorm monitor in the Analgesic region.

**Figure 12:**
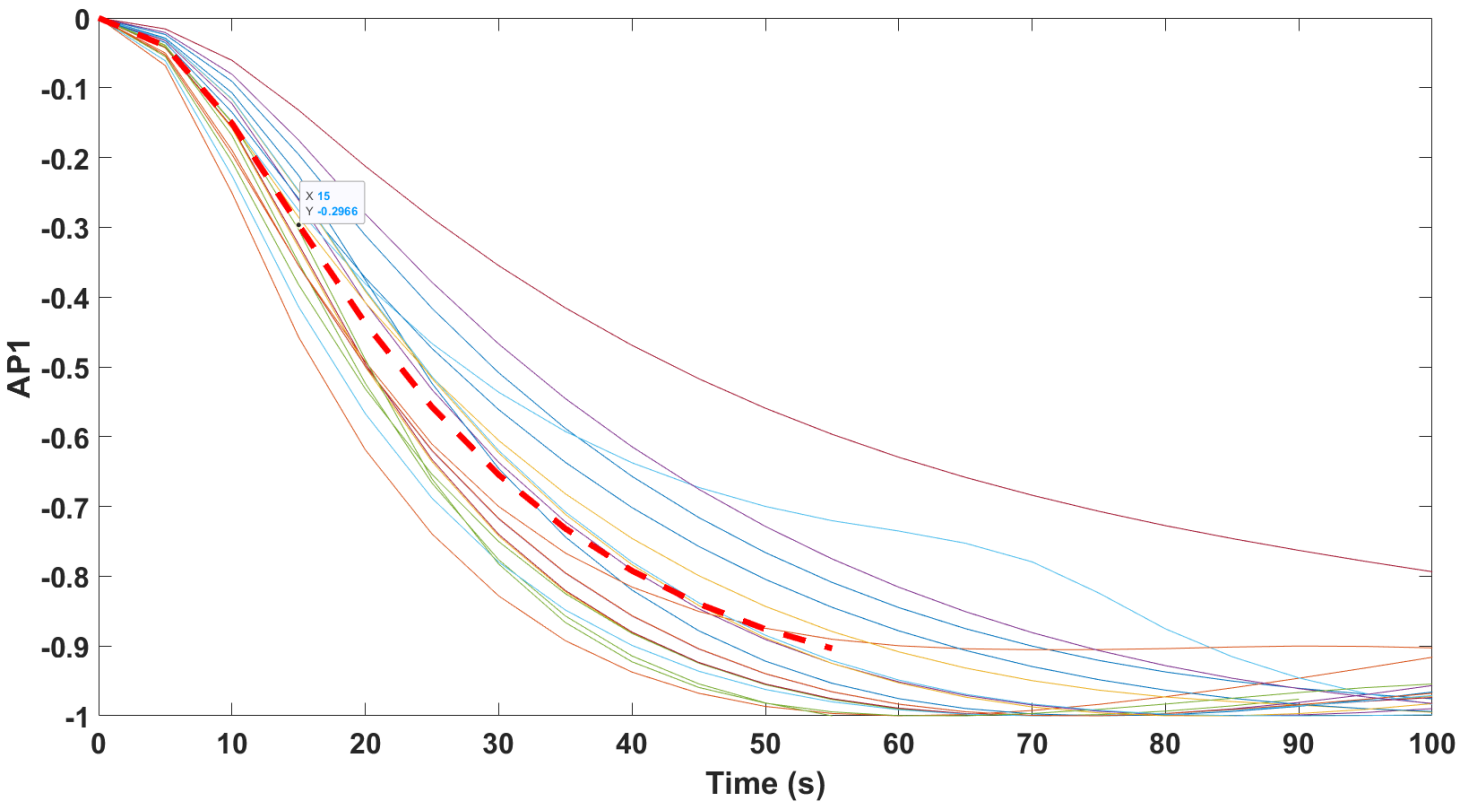
Normalized step responses for all transfer function models identified from patients evaluated with the AnspecPro monitor in the Analgesic region for index AP1.

**Figure 13:**
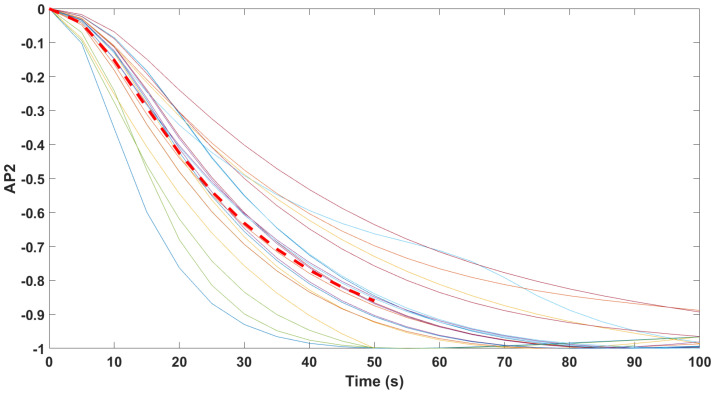
Normalized step responses for all transfer function models identified from patients evaluated with the AnspecPro monitor in the Analgesic region for index AP2.

**Figure 14:**
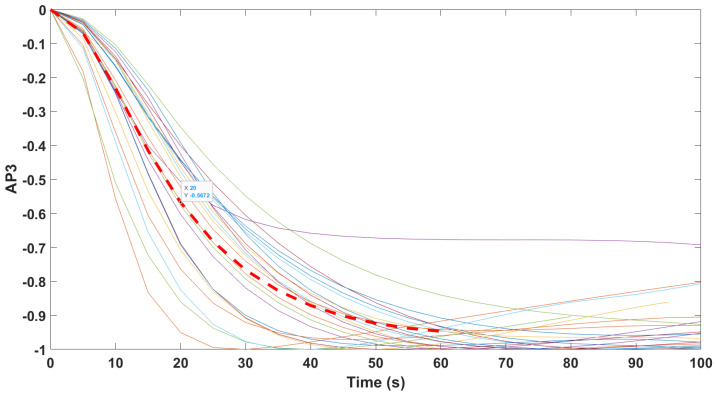
Normalized step responses for all transfer function models identified from patients evaluated with the AnspecPro monitor in the Analgesic region for index AP3.

**Figure 15:**
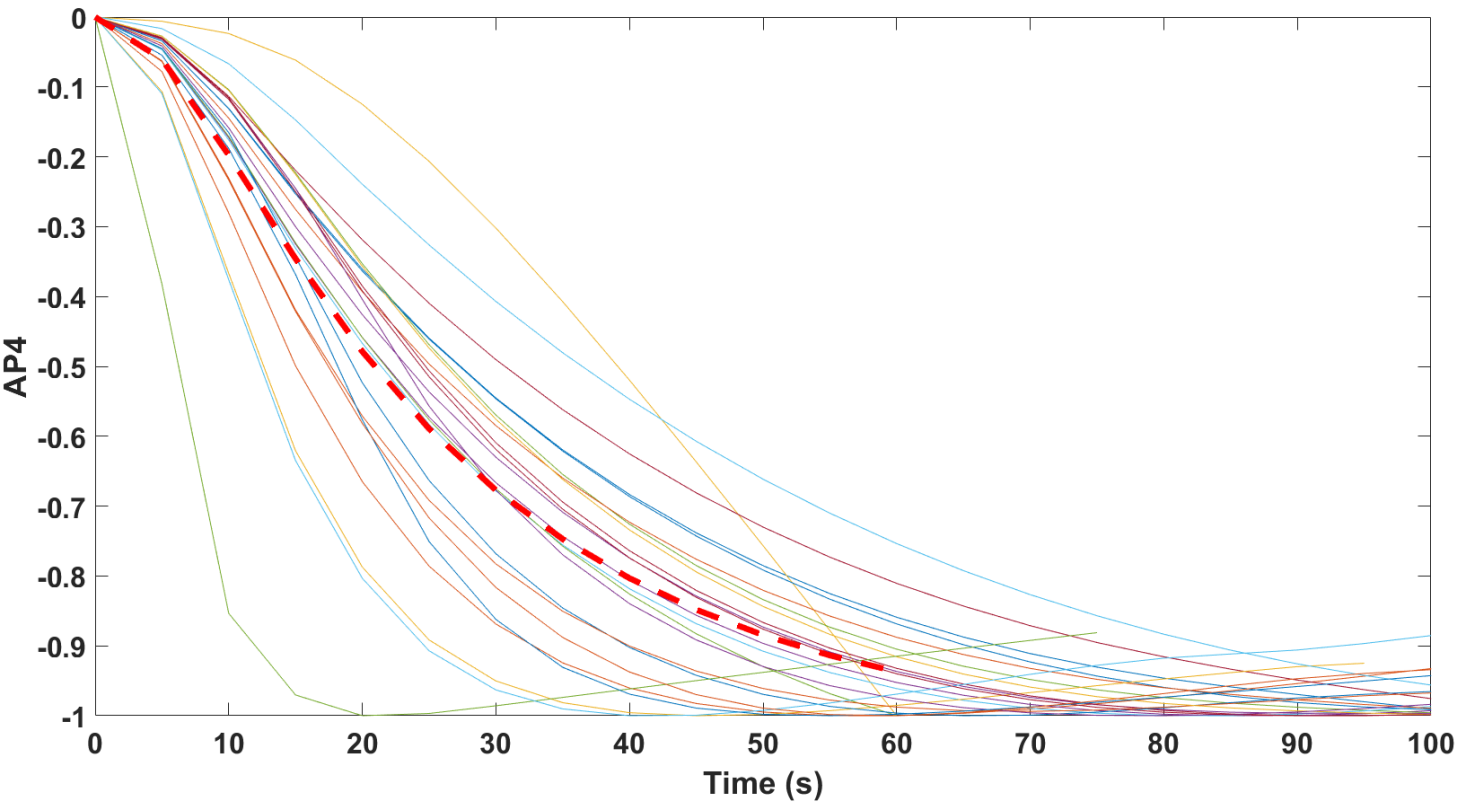
Normalized step responses for all transfer function models identified from patients evaluated with the AnspecPro monitor in the Analgesic region for index AP4.

Finally, the mathematical relationship in an input (i.e., Remifentanil) to output (i.e., N/AN index) context of a dynamic system (patient) is the transfer function model [49]. The transfer function models identified from the index data for all monitors are summarized hereafter. For each patient, the normalized step response from the identified model is given in Figure 10 for the Medasense monitor, in Figure 11 for the Medstorm monitor, and in Figure 12, Figure 13, Figure 14 and Figure 15 for the AnspecPro monitor. The structure of the TF is $K/\left(s+\tau_{1}\right)\left(s+\tau_{2}\right)$, and in Table 3, we give the coefficients for the averaged transfer function models along with their standard deviations. Further details are provided in the Supplementary Material file.

## 4. Discussion

For the first time, the prototype skin-impedance-based monitor AnspecPro was evaluated in patients in general anesthesia. An index of nociception/anti-nociception (N/AN) was defined from the raw skin impedance data. In fact, several indexes were proposed, to broaden the selection range of those best suited for a patient’s state evaluation. Each of these indexes resulted from physiologically-based parametric models, and they capture different biotissue properties and afferent variability in response to drug input and/or nociception stimulation.

The nociceptive stimulus test applied with the standard TOF (train of four) test delivered useful information for concluding that the prototype device AnspecPro is able to detect changes in skin impedance as a result of noxious stimulus. Its degree of sensitiveness to these variations produced an effect that was not statistically different from the N/AN index in the commercial devices (p>0.5). With respect to evaluation of the four indices developed for the AP device, there was also no statistical significant difference (p<0.162) (Figure 9). All three monitors were sensitive to the TOF test applied as nociceptive stimulus.

The TOF is a standard pain stimulus in awake patients, and it is a standard nociception pain stimulus. Unlike other stimuli, it has the advantage of being repetitive (always the same intensity and same time of duration), which makes it a good objective test. The standard tetanic stimulation used to evaluate nociception levels is standardly applied on the handpalm, but it was not possible in our case as our nociception monitors have sensing patches on both hands, and therefore may interfere with the electric signals from the devices. On the other hand, a tetanic stimulus is not ethically acceptable for application on the forehead of a patient.

Hence, we chose to apply the 3xTOF stimulus to the forehead of the patient. This is an extremely painful stimulus in awake patients, so it surely also has a relatively high intensity in anesthetized patients, making it relevant for our check test here.In our study, the TOF was given in a set of three TOFs, one after another, which is surely a large intensity for testing on patients and monitoring by the devices we analyze here.

It is true that when TOF is applied in patients under only Propofol-induced hypnoses, this may not be very intense and probably not as intense as the surgical stimulus; but we cannot know this beforehand as there is large uncertainty regarding the surgical stimulus as well as in terms of its intensity, as it is also not as repetitive as TOF.

When comparing (see Supplementary Material) statistical analysis of the regions before and during the standardized TOF, there was a difference between the two states in all devices and for all indices, namely, a decrease in values during the TOF test data. Note that all devices measure a dose-related corresponding effect; in other words, it is a measure of the effect of the drug potency on the specific patients in the group. Our comparison results indicate that there is an increase in the effect resulting from the potency of the drug (i.e., dose effect) because Propofol and Remifentanil were administered (see Supplementary Material). From a control engineering point of view, as well as from a closed-loop control of anesthesia perspective, this result is very logical and makes sense to all coauthors from both clinical and engineering disciplines. Moreover, it is a consistent and visible result in all devices. From a control engineering perspective, the TOF is a disturbance (or, in clinical terms, it is a stimulus). A decrease in the effect of the drug indicates that more of the drug should be applied to maintain the same effect in presence of stimulus/disturbance.

If all devices consistently have the same “reaction” to the “disturbance” given by TOF, it means that all of them are sensitive to capturing the effect of stimuli and are, therefore, suitable candidates for closed-loop control of anesthesia involving both humans and computers in the decision-making process of finding optimal infusion rates.

The parametric model from (3) is based on the distributed parameter properties of an electrical network equivalent for skin layer impedance measurements. This is mainly capacitive in properties with a scaling factor in terms of resistance coming from the biological tissue viscoelastic properties [50]. The threshold of neuronal signaling in the sodium–potassium pump involved in gate mitigation for electrical stimulus from sensorial to perceptual nodes within the pain pathways [37] is modified by the electrical transmissibility of the tissue in response to molecular binding of the administered opioid. This is represented by the variations in the capacitive values of the recurrent ladder network herein proposed. It follows that the index AP1 from (4) is able to capture variations in the capacitive sensitivity of tissue with changes in its electrochemical properties. The recurrent ratio of the capacitance values will affect the slope of the Bode characteristic of the frequency response of such a dynamic intricate process and, in turn, will affect the bandwidth of the process (i.e., it affects the dynamic response to drug or stimulus input) [39,49]. By analogy to our in silico studies [33], this index characterizes the variability of electrochemical properties of tissue impedance.

Physiologically similar, but in a more compact form as it has fewer parameters, is the model from (6). The deep link to tissue electrochemical properties is lost here due to simplification of the model structure, but it maintains the characterization of the dynamic response. This is through the pole–zero locations which are identified in the Nyquist complex plane, i.e., the roots of the polynomials of this model [39]. The index AP2 will capture any variations in the pole–zero locations, i.e., again, related to changes in the dynamic response of the tissue and properties such as electrical conductivity.

The lumped fractional order impedance model from (8) has a physiological interpretation as well; namely, it is the equivalent representation of tissue as a Debye or dielectric material, and the index AP3 is the relaxation time constant with a scale factor K. This particular physiological interpretation is very suitable for properties of tissue with opioid as it is equivalent of dispersion phenomena, closely related to diffusion. The effect of opioid in molecular binding progression tends to behave as Debye first-order model dispersion. However, the electrical dispersion characteristics assume a uniformly lossy material (i.e., tissue) and this might not be true as the pharmacokinetic models for drugs to take effect are compartmental-based models, i.e., a central compartment (blood) and two peripheral compartments (muscle and fat). Still, permittivity and conductivity are properties well encompassed by Debye circuit models, of which the model (8) is a simplified version [51]. Again, these properties will vary with changes occurring in the sodium–potassium pump as a result of an opioid or of an noxious stimulus presence.

For a two-layered material Debye model, the relaxation time constant is given by the formula:

(11) $$\tau =\epsilon_{0}\epsilon_{r1}\frac{1}{(\sigma_{1}+\sigma_{2})/2}$$

where $\epsilon_{0}$ is the residual permittivity of the material in suspended form (no input), $\epsilon_{r1}$ is the permittivity of the material in the first layer, and $\sigma_{1}$ and $\sigma_{2}$ denote the conductivity in the first and second layer of material, respectively. For multilayered materials, such as in the biological tissue case, the β is related to the relative permittivity between the layers, and it affects the relaxation frequency of the material [51]. The term *p* is related to the relative conductivity of electrochemical properties between layers. The index AP4 could be indicative of relative changes in permittivity and conductivity of the tissue.

All indexes have physiological bases and seem to be correlated to changes in Remifentanil administration. As no statistical significant difference is observed, the choice of a singular index is, therefore, difficult. From the correlation point of view, the indexes AP2 and AP3 seemed best correlated, with a prevalent physiological basis in AP3 index.

All transfer function models gave similar time constant around 1 min for reaction time to Remifentanil infusion, which corresponds well to time constants found in the central compartment (i.e., blood). Interpatient variability was well observed in the identified transfer function models for all monitors and for all indices, which suggests that both the CE labeled monitors of Medasense and Medstorm gave similar performance to the prototype monitor AnspecPro.

The use of AnspecPro as an N/AN monitor or as a monitoring device for the N/AN state of the patient under general anesthesia is suitable, under the assumption that it performs similar to the commercial devices used here for comparison. It is worth mentioning that, of the three devices, the Medstorm device had the poorest performance in terms of signal quality index, explainable by the fact that the algorithm to extract the SC index is based on a single sinusoid and single conductance information variations of intensity over time. By contrast, the NOL index from the Medasense device was most reliable in terms of signal quality and had also the highest correlation among the devices to variations in Remifentanil infusion profiles in the Analgesic region of our protocol. This is explainable by the fact that it uses a multivariate algorithm on several signals and, thus, collects more information from the patient to extract the N/AN level. As such, the prototype AnspecPro uses a multisine to extract skin impedance variations over time and frequency, but no other signal from the patient. It is, therefore, more complex than the Medstorm device and less complex than the Medasense device. Both the NOL and AP devices are suitable for closed-loop control, with preference given to NOL for being a multivariate signal processor of the patient’s state.

## 5. Conclusions

This paper introduces the validation of the AnspecPro monitor as a nociception/anti-nociception monitor under general anesthesia, as well as a comparison analysis of three monitors. Two commercial pain monitors have a CE label and one is a prototype device. All monitors perform similar under well-designed protocol suitable for fair comparison, concluding that the prototype device detects nociception with similar performance to the commercial ones. As a perspective for the application of closed-loop control of general anesthesia, the AnspecPro monitor and its multifrequency impedance analysis could be well paired with the NOL index to overall improve the N/AN state evaluation in patients under general anesthesia. 

## Figures and Tables

**Table 1:** Patient biometrics and monitor selection (age in years, height in cm, weight in kg, and gender X denotes transgender).

No	Monitor 1	Monitor 2	Gender	Age	Height	Weight	Surgery
1	AnspecPro	Medasense	F	31	163	49	Varicetomy
2	Medstorm	Medasense	F	44	168	57	Arthroscopy
3	Medasense	AnspecPro	F	42	168	70	Varicetomy
4	AnspecPro	Medasense	F	71	158	69	Varicetomy
5	Medasense	Medstorm	F	38	182	87.6	Anal fistula
6	Medstorm	Medasense	F	41	175	135	Hemorrhoids
7	Medasense	Medstorm	F	68	172	67	Laparoscopic cholecystectomy
8	Medstorm	AnspecPro	M	59	183	76	Inguinal hernia
9	Medstorm	AnspecPro	M	50	186	96	Laparoscopic inguinal hernia
10	Medasense	AnspecPro	M	73	181	83	Laparoscopic inguinal hernia
11	AnspecPro	Medstorm	X	22	165	87	Hysterectomy
12	Medstorm	Medasense	M	29	183	92	Laparoscopic inguinal hernia
13	AnspecPro	Medstorm	F	54	160	48	Laparoscopic hysterectomy
14	Medstorm	AnspecPro	X	19	168.7	59	Laparoscopic hysterectomy
15	AnspecPro	Medasense	X	26	155	61	Laparoscopic hysterectomy
16	Medstorm	Medasense	F	54	163	58	Abdominal hysterectomy
17	AnspecPro	Medstorm	F	50	174	64	Breast augmentation & Liposuction
18	AnspecPro	Medasense	F	30	176	74	Unilateral open inguinal hernia
19	Medasense	Medstorm	F	57	164	70	Hysterectomy
20	Medasense	Medstorm	X	33	162	80	Laparoscopic hysterectomy
21	Medasense	AnspecPro	F	62	168	88	Exploratory laparotomy, Colpopromontoriopexy, placing JJ bilateral
22	Medstorm	AnspecPro	F	48	155	56	Hysterectomy
23	AnspecPro	Medstorm	M	62	183	85	Varicetomy
24	Medstorm	AnspecPro	F	36	168	63	Laparoscopic myomectomy
25	Medasense	AnspecPro	M	58	179	94	Inguinal hernia laparoscopy
26	AnspecPro	Medstorm	F	65	162	87	Gallbladder removal
27	Medstorm	Medasense	F	49	167	86	Laparoscopic cholecystectomy + Cholangiography.
28	Medstorm	Medasense	M	46	187	97	Laparoscopic bilateral inguinal hernia
29	AnspecPro	Medasense	M	68	176	85	Laparoscopic cholecystectomy + Cholangiography.
30	Medasense	Medstorm	F	50	167	70	Abdominal hysterectomy
31	AnspecPro	Medasense	M	54	175	90	Laparoscopic inguinal hernia unilateral
32	AnspecPro	Medstorm	F	64	164	96	Laparoscopic hysterectomy
33	Medstorm	AnspecPro	F	46	170	82	Laparoscopic hysterectomy
34	AnspecPro	Medasense	F	58	157	53.5	Adnexektomie laparoscopic bilateral
35	Medstorm	AnspecPro	F	41	167	65	Laparoscopic hysterectomy
36	Medasense	AnspecPro	F	40	159	63	Vulvectomy + Laparoscopic hysterectomy
37	AnspecPro	Medasense	F	30	160	53	laparoscopy Chromotubation + Operative + gynecological endometriosis
38	Medstorm	AnspecPro	F	18	163	64	Remove O.S. Material + Fulkerson Osteotomy ORTHO
39	Medasense	AnspecPro	F	64	158	58	Exploratory laparotomy
40	Medstorm	AnspecPro	M	63	171	104	Scrotal exploration
41	Medstorm	AnspecPro	F	30	156	44	Diagnostic hysteroscopy + laparoscopy diagnostic + Chromotubation
42	AnspecPro	Medstorm	F	19	168	54	Diagnostic hysteroscopy + laparoscopy diagnostic + Chromotubation
43	AnspecPro	Medasense	F	51	168	58	Adnexektomie laparoscopic bilateral
44	Medasense	Medstorm	F	59	171	75	Vaginal hysterectomy
45	AnspecPro	Medstorm	M	73	174	67	Urethroplasty
46	Medstorm	Medasense	M	23	173	66	Circumcision
47	AnspecPro	Medstorm	M	25	181	74.5	Circumcision
48	Medstorm	AnspecPro	M	31	170	85	Circumcision
49	Medasense	Medstorm	M	32	186	100	Resection of scrotal injury
50	AnspecPro	Medasense	F	40	157	85	Laparoscopic hysterectomy
51	Medstorm	Medasense	F	27	157	58	Laparoscopic cystectomy
52	Medasense	Medstorm	F	71	174	71	Anterior and Posterior colporrhaphy
53	AnspecPro	Medasense	M	32	180	78	Vaso vasostomie
54	Medstorm	AnspecPro	F	21	166	63	laparoscopic ovariectomy
55	AnspecPro	Medstorm	X	48	170	84	Laparoscopic hysterectomy
56	Medasense	AnspecPro	M	63	168	82	Bilateral open inguinal hernia
57	AnspecPro	Medstorm	M	71	175	66	Laparoscopic bilateral inguinal hernia
58	AnspecPro	Medasense	F	48	165	76	Laparoscopy exploration gyneco
59	Medstorm	AnspecPro	F	71	167	83	Colpopromantoriopexia laparoscopy
60	Medstorm	Medasense	X	21	160	60	Laparoscopic hysterectomy
61	AnspecPro	Medasense	X	21	171	73	Laparoscopic hysterectomy
62	Medstorm	Medasense	X	34	172	90	Laparoscopic hysterectomy
63	Medstorm	AnspecPro	X	26	170	50	Laparoscopic hysterectomy
64	Medasense	AnspecPro	F	33	172	94	Resection of ovarian cyst
65	AnspecPro	Medstorm	X	22	172	69	Laparoscopic hysterectomy
66	Medstorm	AnspecPro	F	70	165	62	Bilateral adnexectomy
67	Medstorm	Medasense	X	21	168	56	Laparoscopic hysterectomy
68	AnspecPro	Medasense	F	35	160	82	Myomectomy
69	Medstorm	Medasense	X	21	165	70	Laparoscopic hysterectomy
70	Medstorm	AnspecPro	X	20	155	49	Laparoscopic hysterectomy

**Table 2:** Results of the Granger causality test. The table reports the causality test returned as valid for the number of patients out of the total number of patients tested with the respective device. Medasense device: 26 patients, Medstorm device: 20 patients, and AnspecPro device: 23 patients.

Device	Causality with Propofol	Causality with Remifentanil
Medasense	22/26	22/26
Medstorm	8/20	9/20
AnspecPro AP1	22/23	23/23
AnspecPro AP2	22/23	22/23
AnspecPro AP3	21/23	18/23
AnspecPro AP4	22/23	18/23

**Table 3:** Averaged transfer function model parameters values.

Index	K $\left(10^{3}\right)$	$\tau_{1}$ $\;(10^{3})$	$\tau_{2}$ $\;(10^{3})$
AP1	−3.96 ± 1.5	9.42 ± 5.5	0.33 ± 0.11
AP2	−0.0018 ± 0.007	9.49 ± 3.97	0.35 ± 0.23
AP3	−0.003 ± 0.0005	9.5 ± 1.16	0.64 ± 0.11
AP4	−0.0012 ± 0.004	9.74 ± 5.826	0.39 ± 0.143
Index	K ($10^{4}$)	$\tau_{1}$ ($10^{4}$)	$\tau_{2}$ ($10^{4}$)
Medstorm	−0.0003 ± 0.0002	2.83 ± 2.317	0.064 ± 0.0236
Index	K ($10^{6}$)	$\tau_{1}$ ($10^{6}$)	$\tau_{2}$ ($10^{6}$)
Medasense	0.0037 ± 0.0193	1.58 ± 1.04	0.001 ± 0.003

## Data Availability

Data are contained within the article.

## Supplementary Materials

The following supporting information can be downloaded at: https://www.mdpi.com/article/10.3390/s24072031/s1.

## Appendix A. Protocol Details

Following the illustration from Figure 1, the following measurements were performed. The first registration by the two monitors (awake, no anesthetics) was performed before induction, when the patient’s state was normal and could be considered the baseline measurement (MON1, MON2). After each monitor registered the data for two minutes, the anesthesiologist induced the anesthesia with hypnotics (Propofol), while the second monitors still registered the patient’s response to this change.

A stable clinical level of hypnosis was reached for each patient, observed through a constant effect-site concentration of Propofol, on the BIS/Neurosense monitors and other clinical response signs from the patient. The second registration (hypnotics) by the two monitors started then and continued for two minutes each monitor (MON2, MON1).

The third registration (TOF) was performed when applying TOF stimulus three times for a total of 20–30 s by the anesthesiologist (MON2, MON1). For thirty seconds after TOF finished, the first registering device continued to monitor the patient’s response. The REG3 for the second monitor started after another thirty seconds, waiting for the patient to return to its initial sleepy state.

Remifentanil infusion was started by the anesthesiologist one minute later after TOF registration finished (MON2). During the induction, first, hypnotics was given, followed by analgesics. This was study-specific, so the design would allow the registration of the monitor during a period of hypnosis without analgesics, but with simultaneous nociceptive stimulus induced by a TOF monitor. Moreover, the muscular blockage was performed intravenously by the anesthesiologist with Rocuronium in case the tracheal intubation was needed. During the induction with analgesics infusion, intubation, or insertion of the laryngeal mask and incision, only one monitor remained connected to the patient and registered the patient’s response (MON2).

Patients were randomized to be monitored by two out of the three monitors under investigation. Three groups were created by blinded selection for each two monitors necessary to make statistical pairing possible, as follows: AnspecPro–Medasense, AnspecPro–Medstorm, Medasense–Medstorm. Each was also divided into two, because the study protocol allows only one of the monitors to record while Remifentanil infusion starts, hence the order of the monitor counts for the correlation with the analgesia level (e.g., AnspecPro– Medasense delivers a different dataset in terms of monitoring period compared to the group Medasense–AnspecPro). The choice of the group (which two monitors) was made by random choice of sealed envelopes.

## Appendix B. Numerical Examples for One Patient

Let us analyze the model from (3). Recall here the form of second-order polynomials in the transfer function:

(A1) $$P\left(s\right)=\frac{\omega_{n}^{2}}{s^{2}+2\zeta \omega_{n}+\omega_{n}^{2}}$$

where *s* denotes the Laplace operator. If we bring the model from (3) into the form above, it follows that the natural frequency for the polynomial in *R* is given by

(A2) $$\omega_{n}{,}_{R}=R\sqrt{r_{R}\left(m-1\right)}$$

with the damping factor

(A3) $$\zeta {,}_{R}=\frac{r_{R}\left(m-1\right)R}{R\sqrt{r_{R}\left(m-1\right)}}=\sqrt{r_{R}\left(m-1\right)}$$

leading to the relation $\omega_{n}=R\zeta$. Similarly, it can be obtained for the polynomials in *C*: $\omega_{n}{,}_{C}=C\sqrt{r_{C}\left(m-1\right)}$, and $\zeta {,}_{C}=\sqrt{r_{C}\left(m-1\right)}$.

For m=1, corresponding to one cell, we have that $M\left(s\right)=\frac{s^{2}}{s^{2}}=1$, with $\omega_{n}=\zeta =0$. For m=2, we have the system:

(A4) $$M\left(s\right)=\frac{s^{2}+2r_{R}Rs+r_{R}R^{2}}{s^{2}+2r_{C}Cs+r_{C}C^{2}}$$

and $\zeta {,}_{R}{,}_{2}=\sqrt{r_{R}}$. For m=3, it follows that $\zeta {,}_{R}{,}_{3}=\sqrt{2r_{R}}$, etc.

The examples of identification reported in paper in Figure 3 and Figure 4 are given for the patient indexed 24 as in Table 1. The results of the model parameter identification from (3) averaged over the 33 samples of impedance data over time are R=137.8, C=242, $r_{R}=35.45$, and $r_{C}=6.75$. For all patients, the value m=13 has been fixed. From these results, it follows that the damping factor in this model is always 1<ζ for all values of m=1…13. This corresponds to the dynamic response of an overdamped system, well supported by physiological insight.

Next, let us see an example for the model from (6). For the identification provided in Figure 3, the model coefficients averaged over the 33 samples of impedance values over time are as follows: $Z_{1}=69,769.51$, $P_{1}=-20,972.17$, $Z_{2}=-2,696,379.43$, $P_{2}=57,746.65$, $Z_{3}=-478.05$, $P_{3}=561.66$, $Z_{4}=103,041.20$, $P_{4}=119,503.37$, $Z_{5}=-8506.34$, $P_{5}=528.62$, $Z_{6}=33,455.10$, $P_{6}=-39,844.88$, and K=258.30.

Finally, for the model from (8), the identification result for the same patient and same impedance data over the 33 samples in time are averaged as follows: R=240.50, K=3903.08, p=1927.40, and β=1.66.
