# Supplementary material for: Development, Validation, and Comparison of a Novel Nociception/Anti-Nociception Monitor against Two Commercial Monitors in General Anesthesia
Source: Sensors (Basel). 2024 Mar 22;24(7):2031. doi: 10.3390/s24072031 (PMC11013864; doi:10.3390/s24072031)
Supplement: Supplementary file 1 [file sensors-24-02031-s001.zip › sensors-2861020-supplementary.pdf]

Development, validation and comparison of a novel nociception/anti-nociception monitor against two commercial monitors in general anesthesia

Supplementary material

We performed multiple pairwise comparisons based on one way analysis of variance (anova1) of the group means to further assess the differences between device responses before and after standardized Train-of-Four (TOF) application. This has been done in Mathworks/matlab software platform, and the dedicated function “multcompare” is explained here:
https://www.mathworks.com/help/stats/multiple-comparisons.html

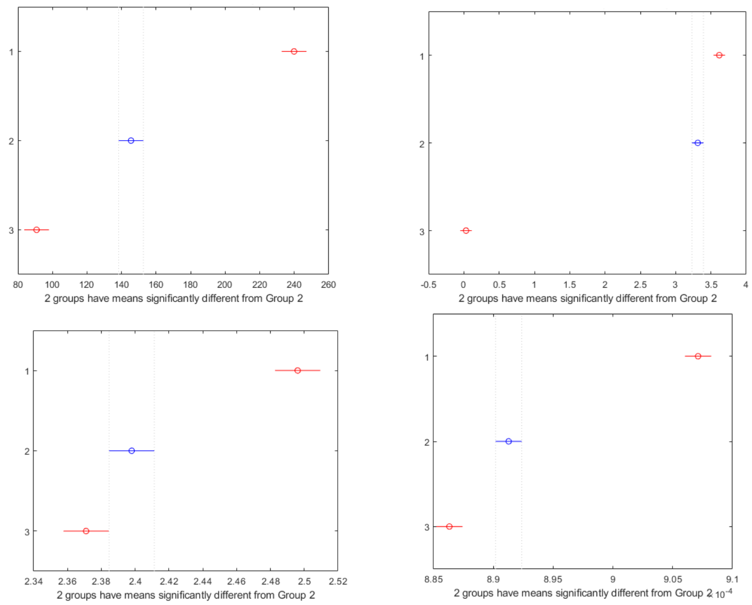

Figure 1: AnspecPro index AP1, AP2, AP3 and AP4 respectively. X-axes denote the corresponding raw values for each signal. Y-axes represent three groups comparison of three regions: Hypnotics(1), TOF(2) and Analgesics(3) region as described in the clinical protocol figure in the paper. We observe consistent response for all indexes in decreased mean values in response to propofol infusion during TOF and to remifentanil infusion during analgesics.

Additional information from data exists for effect of surgical stimulus, but the surgical stimulus is not a controlled stimulus nor in amplitude, neither in duration. This is also varying as per patient respective surgery type and other factors arising during surgery. It makes no sense from an engineering point of view to compare apples and pears by using surgical stimuli region available in our data.

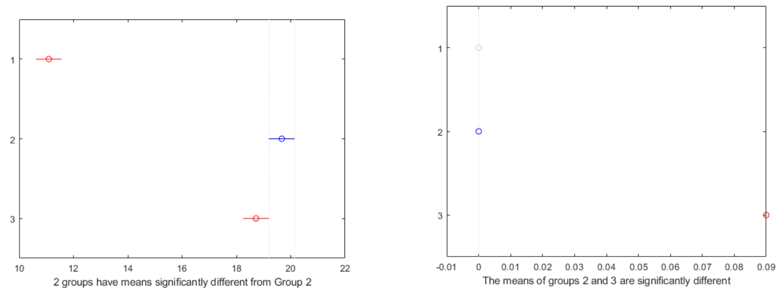

Figure 2: Propofol (left) and Remifentanil (right) infusion rates when AnspecPro is used as monitor 2. X-axes denote the corresponding raw values for each signal. Y-axes represent three groups comparison of three regions: Hypnotics(1), TOF(2) and Analgesics(3) region as described in the clinical protocol figure in the paper.

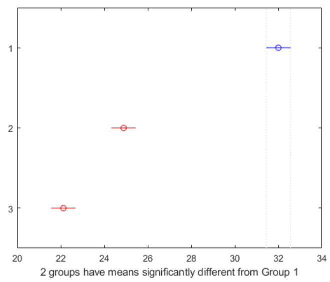

Figure 3: NOL index. X-axes denote the corresponding raw values for each signal. Y-axes represent three groups comparison of three regions: Hypnotics(1), TOF(2) and Analgesics(3) region as described in the clinical protocol figure in the paper. We observe consistent response for all indexes in decreased mean values in response to propofol infusion during TOF and to remifentanil infusion during analgesics.

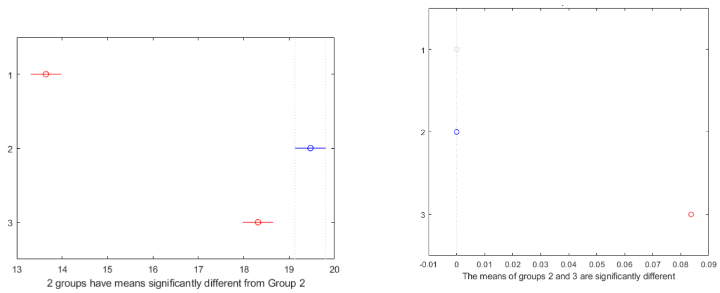

Figure 4: Propofol (left) and Remifentanil (right) infusion rates when Medasense is used as monitor 2. X-axes denote the corresponding raw values for each signal. Y-axes represent three groups comparison of three regions: Hypnotics(1), TOF(2) and Analgesics(3) region as described in the clinical protocol figure in the paper.

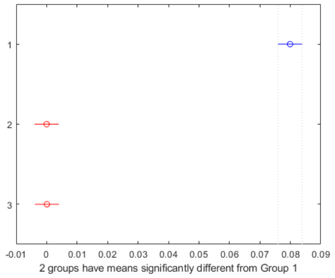

Figure 5: Skin conductance (SC) index. X-axes denote the corresponding raw values for each signal. Y-axes represent three groups comparison of three regions: Hypnotics(1), TOF(2) and Analgesics(3) region as described in the clinical protocol figure in the paper. We observe consistent response for all indexes in decreased mean values in response to propofol infusion during TOF and to remifentanil infusion during analgesics.

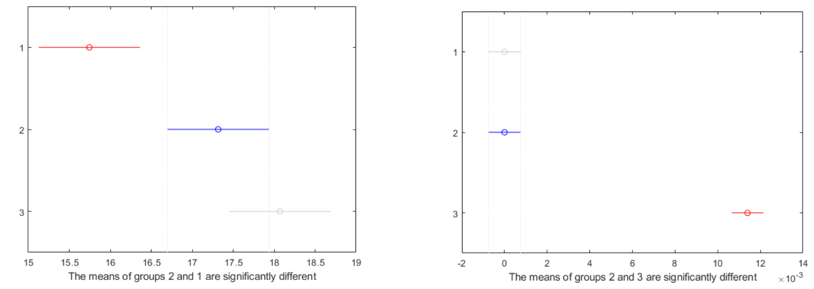

Figure 6: Propofol (left) and Remifentanil (right) infusion rates when Medstorm is used as monitor 2. X-axes denote the corresponding raw values for each signal. Y-axes represent three groups comparison of three regions: Hypnotics(1), TOF(2) and Analgesics(3) region as described in the clinical protocol figure in the paper.

Anderson-Darling test: *h:* Hypothesis test outcome (0 = null hypothesis not rejected; 1 = null hypothesis rejected). *p:* p-value, which is the probability of observing the test statistic at least as extreme as the one observed under the null hypothesis. A commonly used threshold for significance is 0.05. *adstat:* Anderson-Darling statistic value, a measure of how well the data follows a particular distribution. *cv:* Critical value for the test. If the test statistic exceeds this value, the null hypothesis is rejected.

T-test: *h:* Hypothesis test outcome (similar to the Anderson-Darling test). *p:* p-value for the T-test. *ci:* The confidence interval offers a span of values that is likely to contain the true population parameter (such as the mean difference between groups) with a specified level of confidence (typically 95%). This interval reflects the accuracy of the sample estimate and the data's fluctuation. *stat:* T statistic value, which measures the size of the difference relative to the variation in the sample data. The tables 1, 2, and 3 provide a set of statistical test results comparing two methods for assessing the significance of results: the Anderson-Darling test and the T-test.

Table 1: Pain indexes

	Anderson-Darling test				T test			
**Index**	***h***	***p***	**adstat**	**cv**	***h***	***p***	**ci**	**stat**
AP1	1	$5.0\ 10^{-4}$	86.7433	0.7511	1	$5.98\ 10^{-54}$	Min: 153.4330<br>Max: 193.9532	tstat: 16.8310<br>df: 729<br>sd: 278.8259
AP2	1	$5.0\ 10^{-4}$	Inf	0.7511	1	0.0458	Min: 0.0132<br>Max: 1.4036	tstat: 2.0004<br>df: 729<br>sd: 9.5678
AP3	1	$5.0\ 10^{-4}$	1.8948	0.7511	1	0	Min:2.3472<br>Max: 2.4095	tstat: 150.0802<br>df: 729<br>sd: 0.4282
AP4	1	$5.0\ 10^{-4}$	71.7949	0.7511	1	0	Min: $0.8895\ 10^{-3}$<br>Max: $0.8995\ 10^{-3}$	tstat: 349.7216<br>df: 729<br>sd: $6.9108\ 10^{-5}$
NOL	1	$5.0\ 10^{-4}$	4.4366	0.7511	1	$2.73\ 10^{-199}$	Min: 21.5806<br>Max: 14.5898	tstat: 42.2278<br>df: 740<br>sd: 14.5898
SC	1	$5.0\ 10^{-4}$	138.5413	0.7509	1	$5.562\ 10^{-25}$	Min: 0.0382<br>Max: 0.0552	tstat: 10.8008<br>df: 603<br>sd: 0.1063

For Anderson-Darling Test, all indices have a hypothesis test outcome (h) of 1, indicating that the null hypothesis has been rejected for all. This suggests that for each index, the data do not follow the assumed distribution. The p-values are all very low, which reinforces the rejection of the null hypothe-

sis. However, the Anderson-Darling statistic (adstat) varies significantly among the indices, indicating different degrees of deviation from the assumed distribution. SC shows the highest deviation from the assumed distribution in the Anderson-Darling test, while AP2 shows an infinite value (which might indicate a calculation overflow or an error). The critical values (cv) are relatively consistent, suggesting that the threshold for rejecting the null hypothesis was similar across all tests.

Similar to the Anderson-Darling test, the hypothesis T-test outcome (h) is 1 for all indices, indicating a rejection of the null hypothesis in the T-test as well. The p-values are again extremely low for all indices, with NOL and SC having particularly small p-values ($2.73\ 10^{-199}$ for NOL and $1.562\ 10^{-25}$ for SC), which points to a very strong rejection of the null hypothesis.

Table 2: Correlation of pain indexes with Propofol

	Anderson-Darling test				T test			
**Index**	***h***	***p***	**adstat**	**cv**	***h***	***p***	**ci**	**stat**
AP1	0	0.5224	0.3215	0.7251	0	0.5285	Min: -0.1900<br>Max: 0.1004	tstat: -0.6404<br>sd: 0.3358<br>df: 22
AP2	0	0.1924	0.4993	0.7251	1	0.0125	Min: -0.2335<br>Max: -0.0315	tstat: -2.7198<br>sd: 0.2336<br>df: 22
AP3	0	0.2580	0.4491	0.7251	1	0.0016	Min: 0.0723<br>Max: 0.2683	tstat: 3.6037<br>sd: 0.2266<br>df: 22
AP4	0	0.2317	0.4675	0.7251	1	0.0244	Min: 0.0172<br>Max: 0.2258	tstat: 2.4163<br>sd: 0.2412<br>df: 22
NOL	0	0.0530	0.7141	0.7238	0	0.1313	Min: -0.0520<br>Max: 0.3728	tstat: 1.5703<br>sd: 0.4791<br>df: 21
SC	0	0.0968	0.5930	0.6979	0	0.1560	Min: -0.0710<br>Max: 0.3902	tstat: 1.5230<br>sd: 0.3630<br>df: 11

None of the indexes rejects the null hypothesis according to the Anderson-Darling test, suggesting that the data for all indexes fits the distribution being tested. However, for the T-test, both AP3 and AP4 reject the null hypothesis, suggesting that there may be a significant difference being tested for these two indexes. AP1, AP2, NOL, and SC do not show significant results in the T-test, indicating no strong difference for these indexes.

Based on the Anderson-Darling test, AP1 and AP2 show evidence of a non-normal distribution, while the other indexes do not. For the T-test, none of the indexes show a significant difference from the normal distribution. For

Table 3: Correlation of pain indexes with Remifentanil

	Anderson-Darling test				T test			
**Index**	***h***	***p***	**adstat**	**cv**	***h***	***p***	**ci**	**stat**
AP1	1	0.0338	0.7911	0.7251	0	0.8916	Min: -0.1192<br>Max: 0.1362	tstat: 0.1378<br>sd: 0.2952<br>df: 22
AP2	1	0.0276	0.8249	0.7251	0	0.7483	Min: -0.1585<br>Max: 0.1156	tstat: -0.3250<br>sd: 0.3169<br>df: 22
AP3	0	0.6441	0.2788	0.7251	0	0.2083	Min:-0.0390<br>Max: 0.1692	tstat: 1.2965<br>sd: 0.2408<br>df: 22
AP4	0	0.9185	0.1814	0.7251	0	0.6508	Min: -0.0765<br>Max: 0.1200	tstat: 0.4589<br>sd: 0.2272<br>df: 22
NOL	0	0.0871	0.6323	0.7251	0	0.0594	Min: -0.0053<br>Max: 0.2515	tstat: 1.9882<br>sd: 0.2969<br>df: 22
SC	0	0.1760	0.4974	0.6979	0	0.2647	Min: -0.1124<br>Max: 0.3700	tstat: 1.1754<br>sd: 0.3797<br>df: 11

Anderson-Darling Test AP1-AP2: Have a relatively low p-values, which supports the rejection of the null hypothesis. AP3 - SC: Have higher p-values, indicating weaker evidence against the null hypothesis. For T-test, all indexes have h=0, suggesting that the sample means are not significantly different from the test mean. All indexes show high p-values, suggesting no significant difference from the test mean.

Probability plots *(probplot)*: *probplot* creates a normal probability plot comparing the distribution of the data in y to the normal distribution. *Probplot* plots each data point in y using marker symbols and draws a reference line that represents the theoretical distribution. If the sample data has a normal distribution, then the data points appear along the reference line

Quantile-quantile plot *(qqplot)*: *qqplot* displays a quantile-quantile plot of the quantiles of the sample data x versus the theoretical quantile values from a normal distribution. If the distribution of x is normal, then the data plot appears linear. *qqplot* plots each data point and draws two reference lines that represent the theoretical distribution.

Hereafter, the results obtained using the *probplot* (figures 7-12) and *qqplot* (figures 13-18) for all related output signals are given. The data points are either close to or follow the reference line well, which is indicative of normal distribution properties. There are some deviations, but these are relatively minor and can be expected.

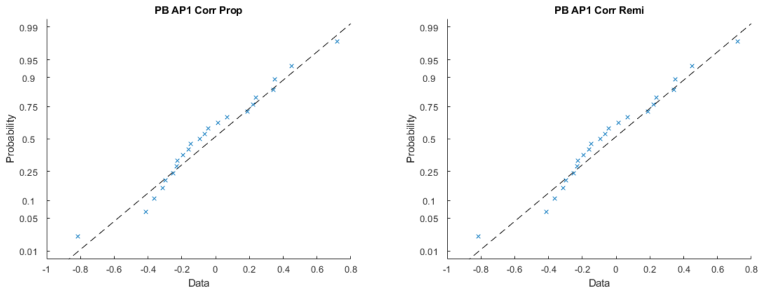

Figure 7: Probability distribution for AP1 index measured from patients in Table 1 (see paper). Left: for Propofol; Right: for Remifentanil

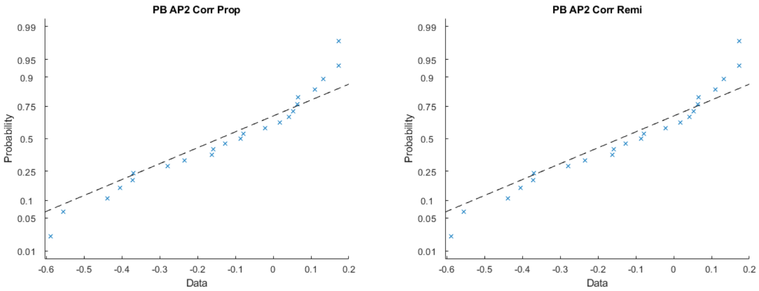

Figure 8: Probability distribution for AP2 index measured from patients in Table 1 (see paper). Left: for Propofol; Right: for Remifentanil

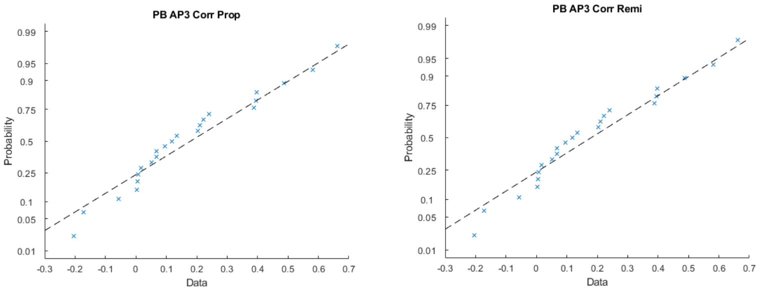

Figure 9: Probability distribution for AP3 index measured from patients in Table 1 (see paper). Left: for Propofol; Right: for Remifentanil

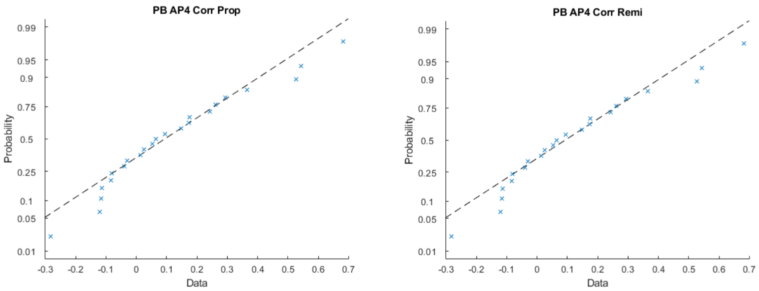

Figure 10: Probability distribution for AP4 index measured from patients in Table 1 (see paper). Left: for Propofol; Right: for Remifentanil

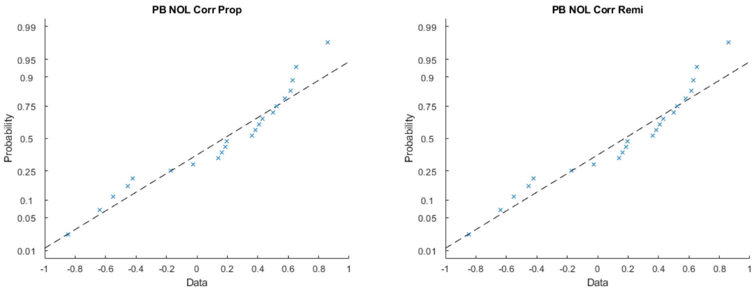

Figure 11: Probability distribution for NOL index measured from patients in Table 1 (see paper). Left: for Propofol; Right: for Remifentanil

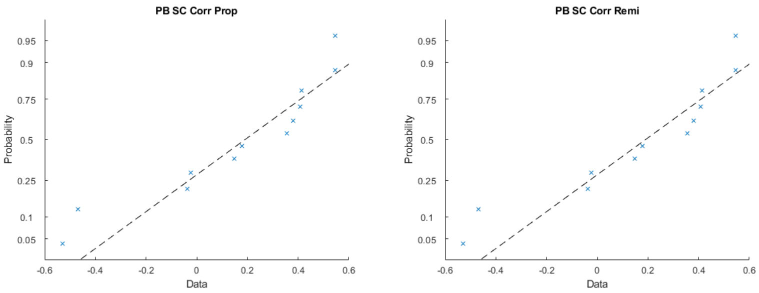

Figure 12: Probability distribution for SC index measured from patients in Table 1 (see paper). Left: for Propofol; Right: for Remifentanil

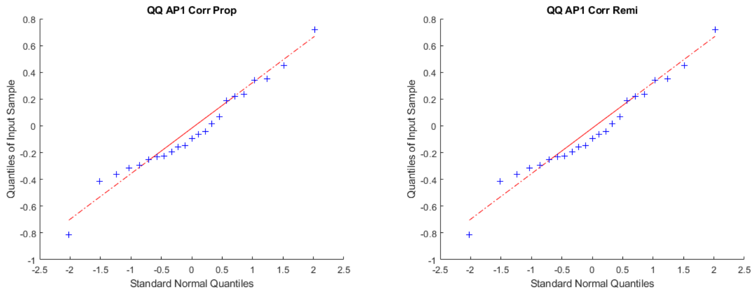

Figure 13: quantile regression plots for the AP1 index measured from patients in Table 1 (see paper). Left: for Propofol; Right: for Remifentanil

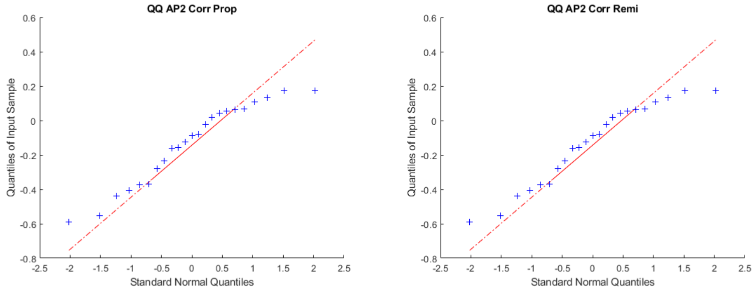

Figure 14: quantile regression plots for the AP2 index measured from patients in Table 1 (see paper). Left: for Propofol; Right: for Remifentanil

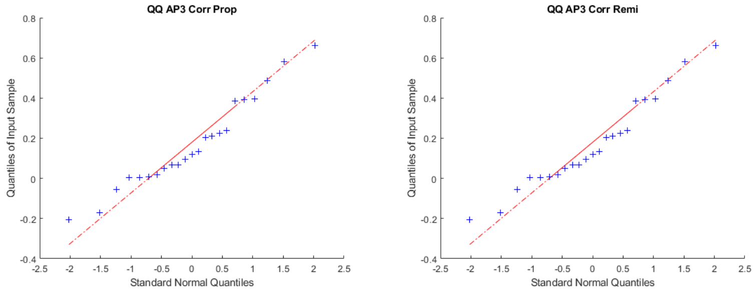

Figure 15: quantile regression plots for the AP3 index measured from patients in Table 1 (see paper). Left: for Propofol; Right: for Remifentanil

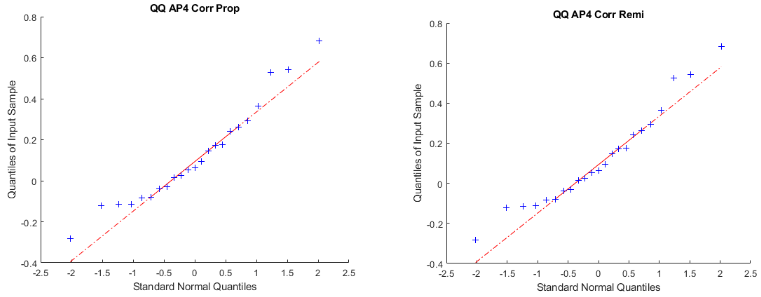

Figure 16: quantile regression plots for the AP4 index measured from patients in Table 1 (see paper). Left: for Propofol; Right: for Remifentanil

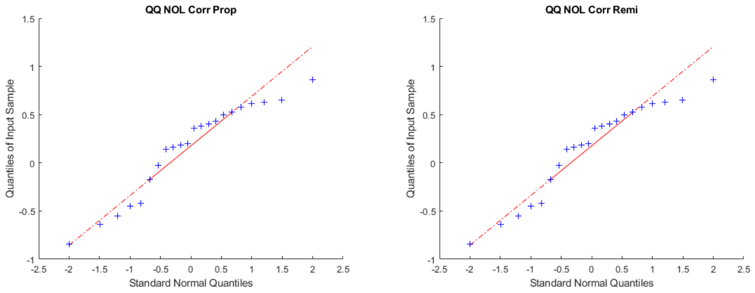

Figure 17: quantile regression plots for the NOL index measured from patients in Table 1 (see paper). Left: for Propofol; Right: for Remifentanil

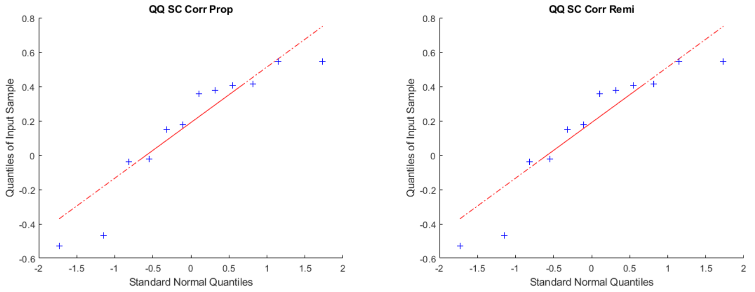

Figure 18: quantile regression plots for the SC index measured from patients in Table 1 (see paper). Left: for Propofol; Right: for Remifentanil
